# Novel suction-based in vivo cutaneous DNA transfection platform

**DOI:** 10.1126/sciadv.abj0611

**Published:** 2021-11-05

**Authors:** Emran O. Lallow, Nandita C. Jhumur, Ijaz Ahmed, Sagar B. Kudchodkar, Christine C. Roberts, Moonsup Jeong, Juliet M. Melnik, Sarah H. Park, Kar Muthumani, Jerry W. Shan, Jeffrey D. Zahn, David I. Shreiber, Jonathan P. Singer, Young K. Park, Joel N. Maslow, Hao Lin

**Affiliations:** 1 Department of Mechanical and Aerospace Engineering, Rutgers, The State University of New Jersey, Piscataway, NJ 08854, USA.; 2 Department of Biomedical Engineering, Rutgers, The State University of New Jersey, Piscataway, NJ 08854, USA.; 3 GeneOne Life Science, Seoul, South Korea.; 4 Graduate School of Biomedical Sciences, Rutgers, The State University of New Jersey, Piscataway, NJ 08854, USA.

## Abstract

This work reports a suction-based cutaneous delivery method for in vivo DNA transfection. Following intradermal Mantoux injection of plasmid DNA in a rat model, a moderate negative pressure is applied to the injection site, a technique similar to Chinese báguàn and Middle Eastern hijama cupping therapies. Strong GFP expression was demonstrated with pEGFP-N1 plasmids where fluorescence was observed as early as 1 hour after dosing. Modeling indicates a strong correlation between focal strain/stress and expression patterns. The absence of visible and/or histological tissue injury contrasts with current in vivo transfection systems such as electroporation. Specific utility was demonstrated with a synthetic SARS-CoV-2 DNA vaccine, which generated host humoral immune response in rats with notable antibody production. This method enables an easy-to-use, cost-effective, and highly scalable platform for both laboratorial transfection needs and clinical applications for nucleic acid–based therapeutics and vaccines.

## Teaser

Suction-mediated cutaneous DNA uptake yielded high in vivo efficiency and thus provides an alternative transfection platform.

## INTRODUCTION

Nucleic acid–based medicine has been extensively developed in the past two decades due to its promise in therapeutics and vaccine (*1*–*4*). Its considerable advantages have been evidently demonstrated in the COVID-19 pandemic where several nucleic acid–based vaccines were rapidly designed, manufactured, and mass-distributed (*5*). Mechanism-wise, synthetic or engineered nucleic acids must enter the host cells and then express and export encoded proteins, e.g., to be processed by the antigen-presenting cells so as to induce an immune response in case of a vaccine. A key step to fulfill their functionality is therefore transfection—the delivery of the nucleic acids to the cytoplasm (RNA) and nucleus (DNA).

Nucleic acid transfection in vivo uses both viral and nonviral platforms. Viral transfection promises efficiency but often faces challenges due to immunogenicity and biosafety concerns. For RNA delivery in vivo, the chemical approach is dominant where nucleic acids are conjugated/encapsulated with polymer or lipid nanoparticles, which both facilitate host cell transfection and protect against degradation by ribonucleases that are prevalent in the cellular environment (*6*, *7*). On the other hand, although DNA is much more stable upon injection, obviating the need for such protection, its delivery to target cells faces, however, a higher barrier. Naked DNA injection typically yields low efficiency, and approaches to enhance transfection include cationic lipids/polymer, electroporation (EP), particle-assisted delivery (gene gun), and pressurized delivery of microdroplets to promote protein expression and immunogenicity, with EP emerging as the most commonly used in recent years (*6*, *8*–*11*). In a recent survey, 47 of 70 clinical trials (from ClinicalTrials.gov, 2010–2017, excluding naked DNA injection) for plasmid DNA–based therapy use EP as the preferred means of delivery (*6*). Current EP devices use a strong electric field of several hundred volts per centimeter to transiently permeabilize the cellular membrane and induce cellular uptake. These electrical pulses can cause muscle contractions, pain, and tissue damage at the site of application and may be contraindicated for those with implantable electrical devices such as defibrillators or pacemakers (*12*–*15*). Most of these devices require substantial training, while additional device costs may also limit the reach into resource-limited regions. The development of alternative delivery systems to address the in vivo transfection bottleneck would therefore help advance synthetic DNA-based therapeutic and vaccines and greatly facilitate rapid responses to future public health crises.

The current work reports a novel, painless, and effective approach for suction-based cutaneous DNA delivery and host-cell transfection that has broad utility in both laboratorial and clinical applications. Following Mantoux injection of DNA into rat skin, a negative pressure is applied to the skin surface atop the injection site. The pressure magnitude is commensurate with that from commercially available open comedone extraction (cosmetic suction) devices in broad human use, and the technique finds analogy in Chinese báguàn and Middle Eastern hijama cupping therapies (*16*, *17*). This simple method induced efficient transfection as determined by green fluorescent protein (GFP) from a GFP-encoding plasmid. This effect echoes prior results of suction-induced transfection of DNA to internal organs, such as the liver and heart performed during open procedures (*18*, *19*), but in a completely noninvasive manner. Furthermore, as a proof of concept for utility, we present a pilot study using a DNA vaccine candidate against severe acute respiratory syndrome coronavirus type 2 (SARS-CoV-2) where strong immune responses were elicited. These results are described below, and on the basis of the strength of these results, our group has advanced this technology into clinical trials of a SARS-CoV-2 vaccine (ClinicalTrials.gov listing NCT 04673149).

## RESULTS

### Intradermal suction delivery induces early transgene expression

**Fig. 1.**
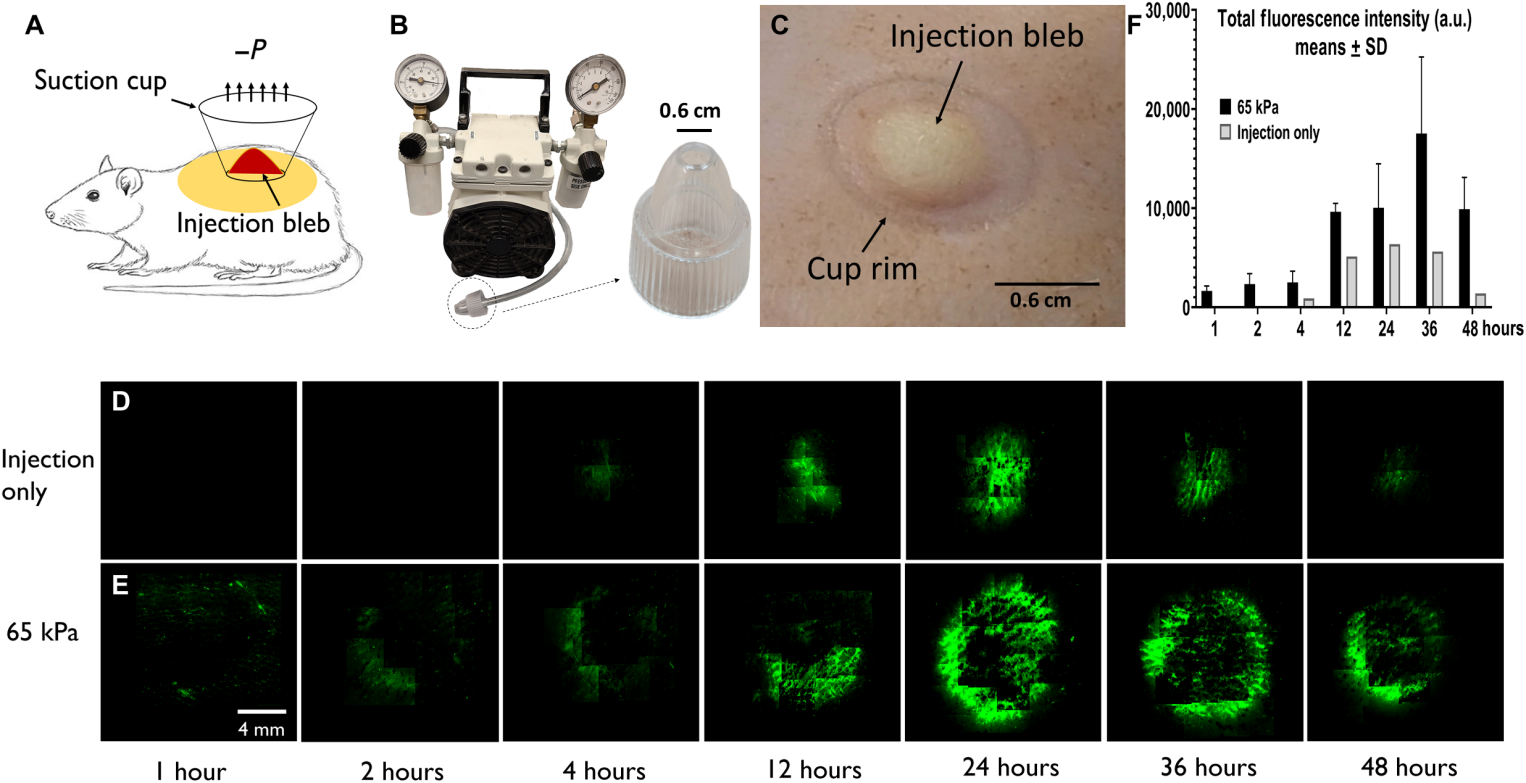
GFP expression under dermal suction. (**A**) Schematic of experimental setup; suction follows Mantoux injection. (**B**) Suction is induced via a disposable cup (6 mm inner diameter at opening) attached to a vacuum pump. (**C**) Skin after suction treatment (65 kPa, 30 s) showed both the injection bleb and the mark by cup rim; no bruising was observed. (**D**) Top view expression pattern for control (injection only) and (**E**) suction-treated skin at 1, 2, 4, 12, 24, 36, and 48 hours after treatment, respectively. (**F**) Top view fluorescence quantification at different time points (*n* = 3). a.u., arbitrary units. Photo credit: Emran Lallow, Rutgers University.

Following Mantoux injection to shaved Sprague-Dawley rat dorsal skin, a negative pressure was applied to the injection bleb (Fig. 1A). The shallow injection targets the epidermal and upper dermal layers and is consistently practiced throughout the current study. Figure 1B demonstrates the setup with an enlarged view of a plastic, disposable, nozzle-shaped suction cup, which connects to a vacuum pump through tubing. The cup had an inner diameter of 6 mm and a rim thickness of 1 mm and completely encompassed the bleb from a 50-μl intradermal (ID) injection. The treated skin area was slightly greater than the cup opening, due to stretching induced by both suction and bleb distention (Fig. 1C).

Suction was applied at 65 kPa for 30 s immediately after injection of 25 μg of pEGFP-N1 plasmid in 50 μl [1× phosphate-buffered saline (PBS)] of solution with internal replicates performed on the same rat at different dorsal sites. Fluorescence microscopy of explanted skin was performed to examine GFP expression with auto- and background fluorescence corrected at the software level by correlating signals from the fluorescein isothiocyanate (FITC)/tetramethyl rhodamine isothiocyanate (TRITC) channels. Injection without secondary applied suction (“injection only,” serving as control) showed detectable expression at 4 hours after delivery and stronger signal at 24 hours (Fig. 1D). In contrast, Fig. 1E shows when injection is followed by suction, punctate GFP expression at the rim was detectable as early as 1 hour after delivery. By 24 hours, expression encompassed most of the cup rim and extended centripetally to the region of displacement. The signal continued to increase with time, and the strongest intensity was observed between 24 and 48 hours. A quantification of the total fluorescence intensity confirmed this trend and is shown in Fig. 1F. Further examination of cryosectioned skin at 24 hours showed a substantial GFP expression level to a depth approximately 400 μm from the skin surface, covering the epidermis and upper dermis. In contrast, injection alone yielded expression confined primarily to a region of 50 μm deep and in the epidermis (fig. S1, A and B). These observations are confirmed by three-dimensional (3D) confocal imaging (fig. S1, C and D, and movie S1). Note that the deeper expression in the dermis has been similarly observed with EP (*20*).

### Expression depends on pressure but not application time, DNA amount, and device type

**Fig. 2.**
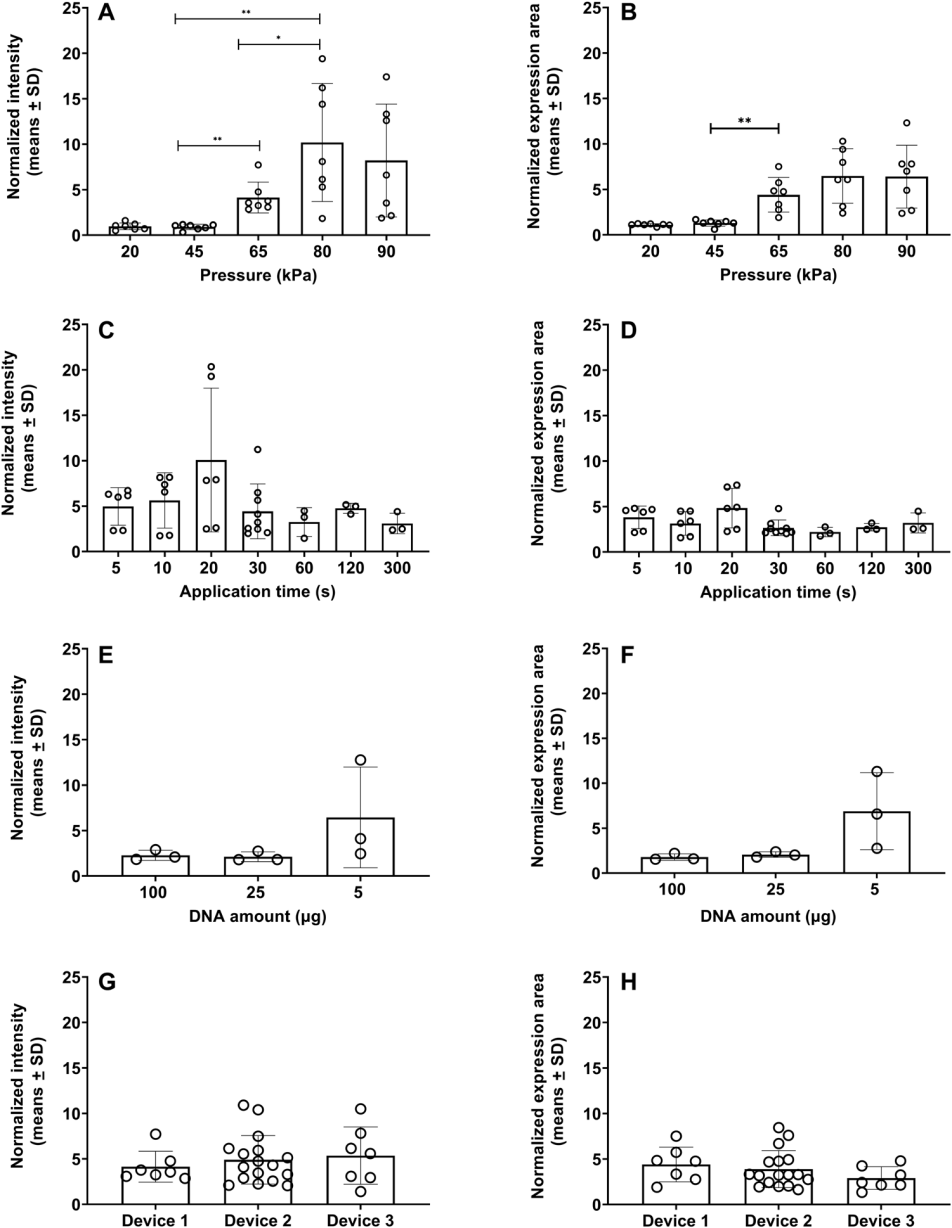
Dependence of expression on pressure, treatment time, DNA amount, and device type. (**A**) Normalized fluorescence intensity and (**B**) normalized expression area as functions of suction pressure; treatment time is 30 s, *n* = 7. (**C**) Normalized fluorescence intensity and (**D**) normalized expression area as functions of treatment time, all at 65 kPa. *n* = 6 for 5, 10, and 20 s; *n* = 9 for 30 s; *n* = 3 for 60, 120, and 300 s. (**E**) Normalized fluorescence intensity and (**F**) normalized expression area for 100, 25, and 5 μg of GFP plasmid in 50 μl of solution, all at 65 kPa for 30 s (*n* = 3). (**G**) Normalized fluorescence intensity and (**H**) normalized expression area across different device types, all at 65 kPa for 30 s. Device 1, *n* = 7; device 2, *n* = 17; device 3, *n* = 7. *P ≤ 0.05; **P ≤ 0.01.

Figure 2 (A and B) presents the effects of suction pressure for suction-assisted DNA entry. The same pEGFP-N1 plasmid solution (25 μg in 50 μl) was injected, followed by a negative pressure application of 20 to 90 kPa for 30 s. The fold enhancement in expression at 24 hours is quantified by first calculating the total fluorescence intensity (Fig. 2A), or the total number of pixels displaying expression signal above background (Fig. 2B) for each injection site, and then normalizing by the respective values from the control location (injection only) within the same animal. At 20 and 45 kPa, the mean values are 0.97 and 0.88, respectively, suggesting essentially no difference relative to control. As pressure increases to 65 and 80 kPa, both mean and scatter increase. At the highest applied pressure of 90 kPa, the mean expression decreases, but the difference with 80 kPa is not statistically significant. Similar trends are shown in Fig. 2B, which is interpreted as effective expression area. Figure 2 (C and D) displays normalized intensity and expression area, respectively, in relation to suction application time ranging from 5 to 300 s, all at 65 kPa. No statistically significant correlation with application time in the 5 to 300 s range was detected.

The enhancement induced by suction is also similar at different DNA amounts except for the lowest value we tested, as demonstrated in Fig. 2 (E and F). For these studies, we apply a pressure of 65 kPa for 30 s, following injections of 2, 5, 25, and 100 μg of pEGFP-N1 plasmid in 50 μl of solutions. Normalized intensity and expression area show no statistical difference in expression for the cases of 5, 25, and 100 μg in total delivered plasmid amount. On the other hand, for the case of 2 μg in 50 μl of solution, no expression was observed either with or without suction following injection, and the negative data are not shown. This result points to a possible threshold of DNA concentration for suction-mediated enhancement to be effective. Not unexpectedly, raw fluorescence intensity does demonstrate a monotonic increase with respect to DNA amount with or without suction, respectively, which is shown in fig. S2.

In addition, quantitatively consistent expression via different negative pressure delivery platforms was observed, provided that the same pressure, suction time, and cup geometry were applied. We have tested three different device setups for suction application, denoting the setup in Fig. 1B as device 1. Device 2 is a commercially available open comedone extraction device for human use. Device 3 is a handheld prototype, delivering a single pressure setting at a single preset duration (see the section ‘Induction of host immune response by suction-mediated DNA vaccine delivery’); the purpose of this design is to demonstrate that the approach can be integrated in an easy-to-use embodiment that requires minimal training. To ensure uniformity, the same suction cup size was used in all three setups. For all cases, the same Mantoux injection of pEGFP-N1 plasmid solution (25 μg in 50 μl) was performed, followed by suction at 65 kPa for 30 s except for the injection-only control. Quantitative results on both normalized intensity and expression area at 24 hours after delivery reveal good consistency across all setups (Fig. 2, G and H).

### Transgene expression pattern is strongly correlated with simulated stress/strain distribution

**Fig. 3.**
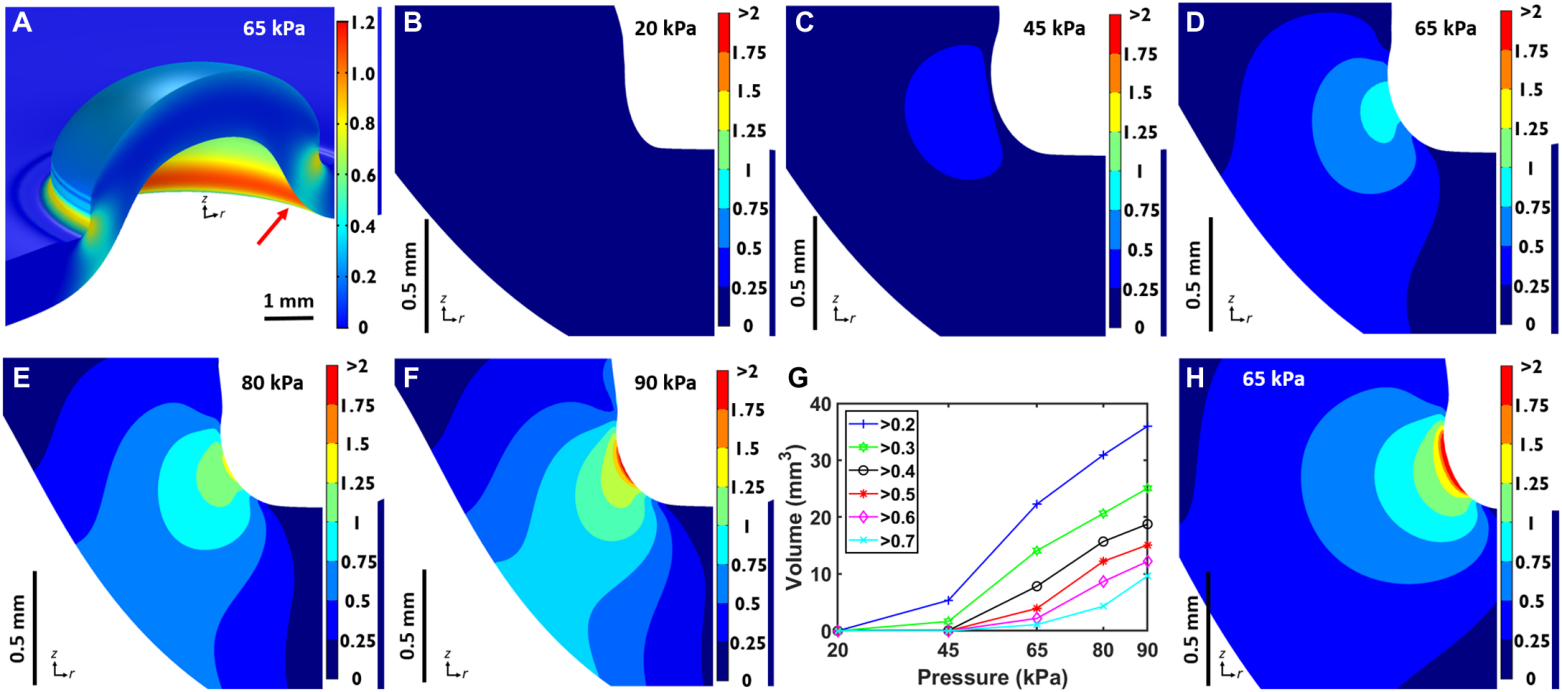
Simulation results. (**A**) 3D view of the color-mapped strain magnitude within the skin layer. (**B** to **F**) Cross-sectional views [in the *rz* plane in the axisymmetric geometry, see arrow in (A)] of the strain magnitude about the focal region at 20, 45, 65, 80, and 90 kPa, respectively. (**G**) Volume quantification within the skin layer for strain magnitude above the various thresholds from 0.2 to 0.7. (**H**) Strain magnitude from simulation using a human skin model at 65 kPa; the cross section shows a thicker skin layer of 2 mm. The strain distribution is quantitatively similar to that within a rat skin despite the anatomical differences.

A numerical simulation is performed to study the stress/strain distribution induced by suction. A multilayer model is used with geometry for the skin, fat, panniculus carnosus (p.c.), and fascia layers based on dissected skin samples of the animals used in the current work (see fig. S3 for a schematic). The simulation is performed in COMSOL Multiphysics with each layer added as a homogeneous, hyperelastic monolith that is axisymmetric around the cup axis (*z* axis in Fig. 3A and fig. S3). Boundary conditions are implemented to emulate the slip contact between the cup rim and the skin under suction, which is observed in the model to expand the application site by 0.2 mm radially to 6.4 mm in diameter. We use a neo-Hookean strain energy to model the skin, fat, and fascia layers (*21*–*25*). Among the many hyperelastic models, this model is the simplest requiring only the Young’s modulus and Poisson’s ratio of the material. Properties for skin are chosen from experimental measurements resulting from a suction chamber that best match the current situation (*22*). Properties for the fascia follow Iatridis *et al.* (*24*), which best represent those of the loose connective tissue. For the p.c. muscle and in the absence of direct measurement results, we choose an Ogden energy function following Bosboom *et al.* (*26*), which demonstrated good agreement between model and experimental data for rat skeletal muscle under in vivo compression. Additional details can be found in the Supplementary Materials.

Figure 3A presents a 3D view of the color-mapped strain magnitude (see Materials and Methods and Supplementary Materials for specific definition) within the skin layer for the exemplary case of 65 kPa in applied pressure; other layers are omitted for clarity. A ring of strain concentration similar to the early time expression pattern created by suction is evident. The same focal patterns are observed for stress and strain energy (Supplementary Materials), and these focal effects appear to arise from the cup rim reacting with a downward force to balance skin lifting due to suction. The depth of this focal ring can be seen to penetrate the entire skin layer (1.2 mm). To further investigate the dependence of deformation on suction pressure magnitude, cross-sectional views of the *rz* plane in the axisymmetric geometry (see red arrow in Fig. 3A) of the strain magnitude about the focal location are shown in Fig. 3 (B to F), where the same color map is used to facilitate comparison. At the lower pressures of 20 and 45 kPa, the strain is mostly under 0.5, with a majority or all of the cross-sectional area under 0.25. At 65 kPa, the area above 0.25 is markedly expanded, and the highest strain reaches up to 1. At 80 and 90 kPa, the trend of strain increase continues, and the area with appreciable strain (>0.5) penetrates the entire skin layer. Figure 3G quantifies the volume within the skin, noting that the simulation is in an axisymmetric geometry, above a threshold strain value. This result is discussed in conjunction with a mechanistic hypothesis below.

### Strain relaxation is a possible trigger for endocytosis

The experimental study suggests that a local threshold pressure is required for transfection activation. In addition, the lack of sensitivity to suction application time suggests that activation does not require prolonged pressure application. Combining these results with the simulation and recent work by Thottacherry *et al.* (*27*), we hypothesize that (i) the molecular uptake is induced by applied deformation and (ii) a relaxation-based endocytotic mechanism is responsible for the uptake. The first hypothesis is supported by the observations above. For the second, Thottacherry *et al.* found that a dynamin independent, clathrin-independent carriers/glycosylphosphotidylinositol-anchored protein enriched compartments (CLIC/GEEC, or CG) endocytotic pathway was rapidly up-regulated in vitro when cells relaxed after being mechanically elongated by a linear strain of 6% for 90 s. The process activates endocytotic reservoir formation as means to recycle excess membrane area and restore membrane tension homeostasis. This finding correlates well with our study where we speculate that suction provides the necessary deformation so as to trigger the CG pathway in vivo. On the basis of comparison of the simulation (e.g., Fig. 3G) with the experiment (e.g., Fig. 2A), a strain magnitude of 0.3 to 0.5 is estimated to be required to generate appreciable transfection. As an order-of-magnitude estimate, if an idealized spherical cell is embedded in this continuum and such strain leads to an incompressible ellipsoidal deformation, then this magnitude of strain translates to an area dilation in the range of 2 to 5% (details on this estimation is included in the Supplementary Materials). This magnitude of area dilation matches well with that in Thottacherry *et al.*, which is estimated to be in the same range (e.g., >2% for an elongated cell of aspect ratio 3 under a linear strain of 6%; see the Supplementary Materials). This pathway is also implicated in EP-mediated molecular delivery, among other possible mechanisms that could include uptake via either clathrin pits, caveolae, and/or Ras-related C3 botulinum toxin substrate 1 (Rac1)–dependent mechanisms (*28*–*30*). Furthermore, similar strain magnitudes can be generated in our simulation of human skin (with different thicknesses and absence of the p.c. muscle; Fig. 3H). This similarity not only suggests that the results will be translatable from the rodent model but also demonstrates the robustness of our model prediction in two completely different anatomical configurations. On the other hand, experiments to further validate the involvement of CG pathway in suction-induced transfection is discussed at the end of the paper.

### Induction of host immune response by suction-mediated DNA vaccine delivery

**Fig. 4.**
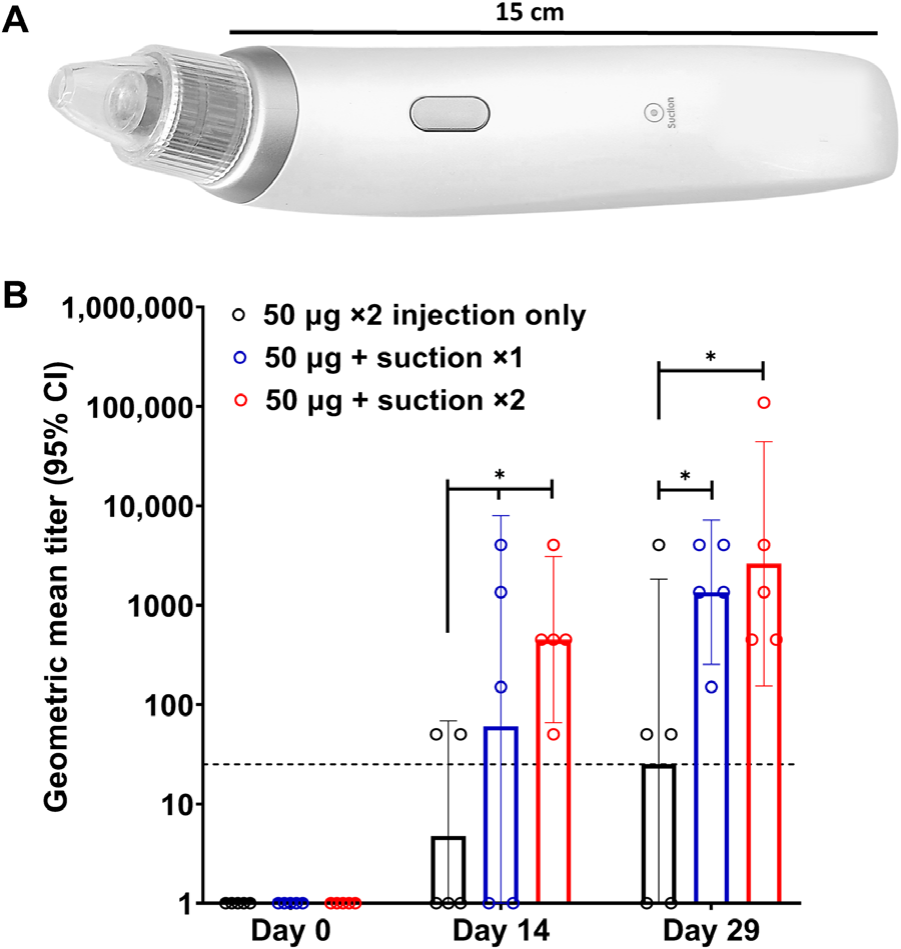
Induction of humoral response to a SARS-CoV-2 DNA vaccine candidate. (**A**) An integrated device used to apply suction for vaccine administration. (**B**) The geometric mean titer (GMT) for IgG against the SARS-CoV-2 S1 protein as determined by ELISA. *n* = 5, *P ≤ 0.05. The bars represent the endpoint titer geometric mean of all five animals for each group; the error bars are the 95% confidence interval (CI). The dashed line at 25 represents the cutoff threshold. Photo credit: Emran Lallow, Rutgers University.

As a proof of concept for utility of the transfection platform, we assessed host immune responses for a candidate DNA vaccine encoding the SARS-CoV-2 spike synthetic DNA delivered by ID Mantoux injection and followed by suction. Device 3 as described above was used, which applied a negative pressure of 65 kPa for 30 s through a cup opening of 6 mm (Fig. 4A). Groups of rats (*n* = 5) were immunized with 50 μg of DNA vaccine in 50 μl of PBS solution. One group received two injections of the vaccine candidate without suction on days 0 and 14 (“50 μg ×2 injection only”); a second group received a single vaccination, followed by dermal suction on day 0 (“50 μg + suction ×1”); and a third group received two vaccinations on days 0 and 14 with both injections followed by dermal suction (“50 μg + suction ×2”). Humoral immune responses to the SARS-CoV-2 S1 spike protein were determined from serum collected before vaccination on days 0 and 14 (2 weeks after first immunization) and then at the time of terminal bleed on day 29 (2 weeks after second immunization). Geometric mean titers (GMTs) of immunoglobulin G (IgG) against the S1 protein as determined by enzyme-linked immunosorbent assay (ELISA) are presented in Fig. 4B. As shown, ELISA titers for humoral responses using the suction device yielded binding antibodies with GMT between 2 × 10^3^ and 5 × 10^3^, significantly greater than injection alone. Injection followed by suction induced seroconversion in 80% of rats by 2 weeks and 100% by 4 weeks. Notably, immune responses in rats who received a single injection followed by suction were not statistically different from those who received two injections, suggesting that this method of DNA vaccine delivery may provide clear benefits.

### Histology indicates no structural damage

**Fig. 5.**
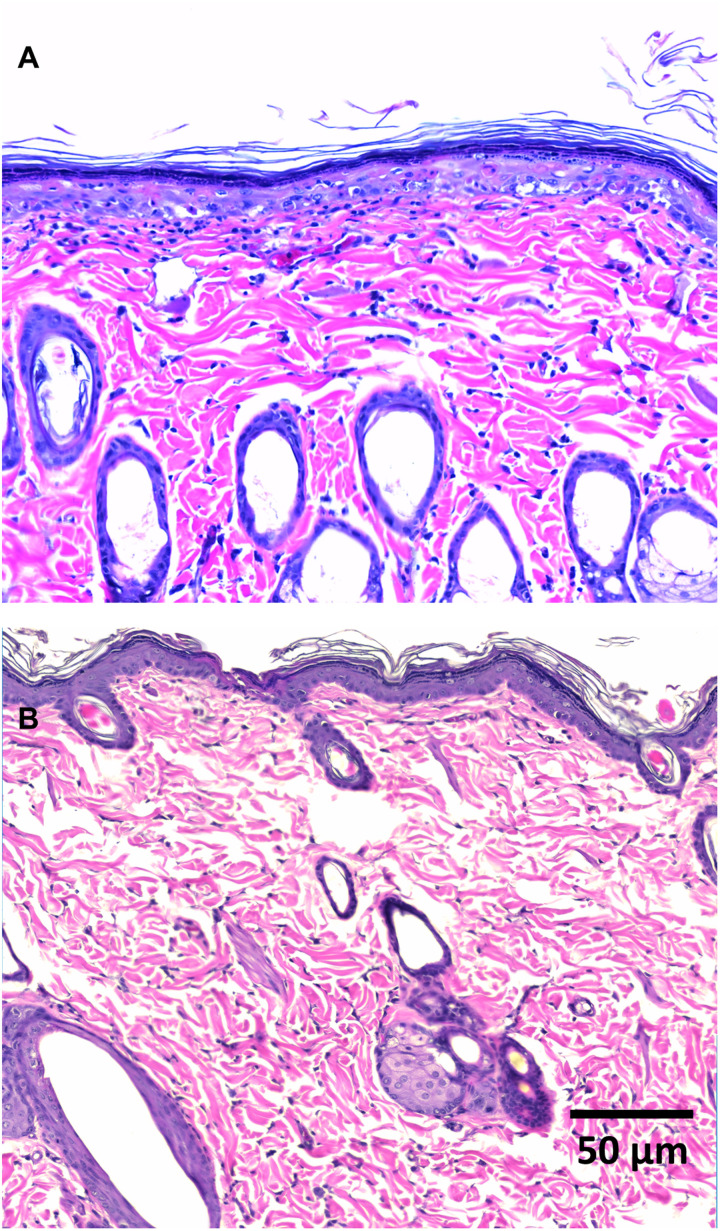
Histology of skin cross sections via H&E staining 24 hours after DNA delivery. (**A**) A representative histological image of a section across the injection bleb from skin that received DNA injection only. (**B**) A representative histological image of a section of a skin area that received DNA injection followed by suction at 65 kPa for 30 s.

Hematoxylin and eosin (H&E) staining was preformed from skin harvested 24 hours after injection of GFP plasmids without suction (Fig. 5A) and followed by suction treatment at 65 kPa, 30 s (Fig. 5B; wider view in fig. S9). No evidence of tissue damage (including structural damage such as layer separation) or lymphocyte infiltration was presented in sections within the injection/suction-treated region, and no remarkable difference was observed when compared against skin that received DNA injection only. Such damage is not only commonly observed for EP but has been posited as necessary to ensure an immune response (*13*, *31*). In contrast, suction is able to achieve the same goal in the absence of histological evidence of damage.

## DISCUSSION

Development of enhanced delivery technologies plays an instrumental role in bringing nucleic acid–based biologics to broad use and clinical relevance. As presented above, we demonstrated an alternative, safe, and effective cutaneous transfection platform that yields high levels of transgene expression. The absence of tissue injury and demonstrable adverse reactions to animal immunization model suggests that this technique will be well tolerated in humans.

The basic mechanism for suction-enhanced transfection in vivo has yet to be elucidated, although the correlation between transfection enhancement to deformation is evident. We have proposed a hypothesis centered around the CG endocytotic pathway, and validation will be pursued in ensuing work. This will mainly involve testing resulted transfection efficiency with down-regulation of the CG pathway using drugs such as ML141 and LG186, analogous to the in vitro counterpart in (*27*). However, carrying out such studies in vivo is inherently more challenging due to the differences and complexities in the pharmacological and biochemical configurations in the latter. This is further limited by the lack of availability and specificity in drugs used for testing. A clear illustration of the molecular mechanism is likely a long-term endeavor. Four decades after the initial use of EP for transfection, a definitive identification of the molecular pathways (even for in vitro) remains elusive, although substantial progress has been made in the past few years, where the involvement of more than a single endocytotic pathway has been indicated (*32*).

Despite the absence of a mechanistic understanding, systematic optimization can be pursued. For example, we may perform further studies to elucidate the lower limit of suction application time, cap geometry, “on” and/or “off” cycling velocity, and any dose effects, which are metrics of practical clinical importance. Combining detailed numerical study and examination of the expression pattern, we may also phenomenologically establish the correlation between strain/stress and expression, so as to help us identify the functional parametric space for transfection. These results will aid in the designs of suction-application protocols both in time and pressure, as well as, e.g., the optimal cup geometry to achieve desired strain or stress field.

We envision that this platform will have broad utility for both laboratorial and clinical applications as a robust method of DNA-encoded biologics in general. From the laboratorial perspective, transfection in vivo on animal models can be practiced with ease, avoiding the expensive instrumentation as required for EP or the biosafety restrictions with viral methods. For clinical applications, advantages include (i) low patient discomfort, (ii) device manufacturing scalability, (iii) minimal requirements for user training, and (iv) cost effectiveness. Additional benefits may also be appreciated, as we observed GFP transgene expression as early as 1 hour after injection. The continued increase in the fluorescent signal and area of expression suggests increased antigen expression over time as well. The very rapid expression indicates that, in addition to vaccine administration, there is potential use to treat diseases that require rapid response. Further studies along these directions would likely provide additional clinical utility, which supports that this approach can be field ready in areas of unreliable power grids during an arising outbreak and against different disease targets for the betterment of human and animal health.

## MATERIALS AND METHODS

### GFP plasmid and SARS-CoV-2 DNA vaccine

pEGFP-N1 DNA plasmid, encoding GFP, and a SARS-CoV-2 DNA vaccine candidate, encoding the S protein, were both provided by GeneOne Life Science (Seoul, South Korea). pEGFP-N1 solution was prepared in 1× PBS at 0.5 μg/μl for all studies except the concentration study; the SARS-CoV-2 DNA vaccine candidate was prepared in the same PBS solution at 1 μg/μl for all experiments. Per injection dose, 50 μl of volume was delivered, totaling pEGFP-N1 plasmids of 25 μg and vaccine of 50 μg, respectively. For the concentration study, 100, 25, 5, and 2 μg of pEGFP-N1 were prepared and delivered in 50 μl of 1× PBS solution.

### Animals

Sprague-Dawley male rats (NTac-SD; murine pathogen free), age 7 to 10 weeks, were purchased from Taconic Biosciences Inc. (Germantown, NY). Rats were housed under controlled conditions (12-hour:12-hour light-dark cycle, room temperature). All animal housing and procedures were in accordance with the guidelines established by the Rutgers University Institutional Animal Care and Use Committee, under protocol IACUC-201800077. For experiments, rats were anesthetized with isoflurane and shaved carefully with hair clippers. Skin was cleaned thoroughly with 70% ethanol and water and was left to dry. No signs of damage were visible on the skin after the preparation procedure. A 28-gauge insulin syringe (Comfort Point, Exelint, Redondo Beach, CA) was used for intradermal introduction of DNA plasmids, using the Mantoux method. Fifty microliters of solution was injected into the skin with the concentrations noted in the previous section. The cup attachment to the vacuum devices was placed on top of the injection bleb ensuring a proper contact. Pressure was applied using various devices (described below) and applied over various times ranging between 5 and 300 s.

### Suction devices

Three different setups to generate a negative pressure were tested. The first one (device 1) uses a Nalgene repairable hand-operated polyvinyl chloride vacuum pump (Thermo Fisher Scientific, Waltham, MA); the second (device 2, Westfy Dematron, Acetex) is a commercially available cosmetic open comedone extraction device sold for human use; the third (device 3) is GeneDerm, an integrated, trademarked device manufactured by Cubist Inc. (Model JM11, South Korea). For all cases, pressure was validated with a MityVac MV4560 pressure gauge (AB SKF, Gothenburg, Sweden) and independently via counterbalancing the negative pressure with weights in small increments. To ensure uniformity, the same suction cup size (outer diameter, 8 mm; inner diameter, 6 mm) was used as taken from device 2 or manufactured for device 1, and the treated area after suction was general slightly greater than the injection bleb as shown in Fig. 1. Consistent results are demonstrated by comparing the performance of the three devices under the same parametric condition (Supplementary Materials). The GFP experiments shown in Figs. 1 and 2 were performed using devices 1 and 2, whereas the vaccination study was performed using device 3 with the consideration of convenient deployment in the clinical setting.

### Imaging and quantification for GFP studies

Rats were euthanized, and skin was excised at defined times after plasmid delivery. For Fig. 1F, a single rat with three suction-treated sites and one injection-only site was euthanized at each indicated time point. Fluorescent images were collected with a 4× objective using both FITC and TRITC channels for the same frame (OLYMPUS 1X80, Olympus, Tokyo, Japan; and ORCA285, Hamamatsu, Bridgewater, NJ). A quantification algorithm was developed using MATLAB (R2018a, MathWorks, Natick, MA). Autofluorescence and background signals from the FITC frames were corrected by shifting each TRITC frame intensity peak signal to match its corresponding FITC frame and then subtracting it. Top view GFP expression images presented in this paper (Fig. 1) are adjusted to the same exposure level and corrected following this approach and had their contrast scale fixed at 0 to 2000 (ImageJ, National Institute of Health, Bethesda, MD) to ensure proper visual comparison. Total fluorescence intensity (e.g., in Fig. 2) was quantified by summing the background-corrected values. For expression area, the number of bright (nonzero-valued) pixels after image correction was counted. Normalization was performed by dividing values from the respective control (injection only) cases, which are always sampled from the same rat to ensure comparability. Sections (fig. S1) were produced by embedding skin samples of ~7.2 mm in diameter in optimal cutting temperature compound (OCT) and then cryosectioning vertically at a thickness of 60 μm and collecting every five sections (CM3050S, Leica, Buffalo Grove, IL). Imaging and background correction followed the same protocol as outlined above. The sections had their contrast scale uniformly specified at 0 to 597 using ImageJ for quantitative consistency.

Confocal laser scanning microscopy (LSM 780, Zeiss, Oberkochen, Germany; and EC Plan-Neofluar 10×, Zeiss, Oberkochen, Germany) was used to produce a *z* stack of 10 slices at ~72-μm thickness per slice. The 3D construction of the GFP expression in fig. S1 (C and D) was created using ImageJ and Zen software, respectively (ImageJ, National Institute of Health, Bethesda, MD; Zen 3.0, Zeiss, Oberkochen, Germany). Movie S1 was created using ImageJ.

### Blood collection

A 25-gauge butterfly needle (Surshield Safety Winged Infusion Set, Terumo, Somerset, NJ) was used to collect 1 to 1.5 ml of blood via a lateral tail vein. Rats were bled on days 0 and 14 before first and second doses of vaccination, respectively. Blood (5 to 10 ml) was collected at the terminal bleed on day 29 via the vena cava. Blood was centrifuged at 10,000 rpm for 10 min (Allegra X-22R, Beckman Coulter, Brea, CA) to separate the serum. Serum was collected, aliquoted, and stored at −80°C. Freeze-thaw cycles were kept minimal to ensure the integrity of the serum.

### Antigen binding ELISA

ELISA plates (Costar 96-well, Corning, Corning, NY) were coated with SARS-CoV-2 spike S1-Fc recombinant protein (1.5 μg/ml; 40591-V02H, Sino Biological, Chesterbrook, PA) in 1× PBS and incubated overnight at 4°C. Plates were washed three times with PBST (0.05% Tween-20 in 1× PBS; Thermo Fisher Scientific, Waltham, MA). Blocking buffer (10% fetal bovine serum in 1× PBS) was added to the plates and incubated at 37°C for 2 hours. Plates were then washed five times with PBST. Serial dilutions of rat sera were added to the plates and incubated overnight at 37°C. Plates were then washed five times with PBST, and 100 μl of rabbit anti-rat IgG (H+L) secondary antibody horseradish peroxidase (Invitrogen 61-9520, Thermo Fisher Scientific, Waltham, MA) at 1:2000 dilution was added and incubated at 37°C for 1 hour. Plates were again washed five times with PBST. O-phenylenediamine dihydrochloride (OPD) substrate (100 μl; SIGMAFAST OPD, Sigma-Aldrich, St. Louis, MO) was added to the plates and left to develop for 15 min in the dark before adding the stopping solution (1 N of sulfuric acid, Fisher Chemical, Thermo Fisher Scientific, Waltham, MA). Absorbance was measured in duplicates at 492 nm in a plate reader for each sample (Infinite M200 PRO, Tecan, Männedorf, Switzerland). GraphPad Prism (9.0.0, GraphPad Software, San Diego, CA) was used to calculate the endpoint titers. Optical density (OD) values for each duplicate sera sample were averaged, and the endpoint titers for each postimmunization sera were defined as the reciprocal of the highest sera dilution that had an average OD_492_ value greater than 2.5 SDs over the average OD_492_ value of the day 0 sera from the same rat. By convention, the cutoff value for the assay was defined as one-half the value of the lowest dilution (1:50), and endpoint titers of 0 were set at the cutoff value for calculation of GMTs of each group.

### Histology

Skin was excised 24 hours after experiments and fixed in 10% neutral buffered formalin (Fisher Chemical, Thermo Fisher Scientific, Waltham, MA) for 2 days before processing for paraffin embedding. Samples were sectioned at 5-μm thickness, and every 20th section was collected. Slides were H&E-stained using the Optik Type 1 H&E stain system (Avantik Biogroup, Pine Brook, NJ) on a standard schedule on a ST5010 Autostainer XL system (Leica, Buffalo Grove, IL). Remaining paraffin was removed in three changes of xylene. Slides were rehydrated through two changes each of 100% and 95% EtOH and then rinsed in water for 1 min. Slides were then incubated for 5 min in hematoxylin solution (Hematoxylin DK, Avantik Biogroup, Pine Brook, NJ) and rinsed in a clarifier for 30 s, water for 1 min, bluing agent for 30 s, and water again for 1 min. This was followed by a 95% EtOH rinse and an incubation in eosin solution (alcohol-based Eosin-PX, with phloxine) for 75 s. The slides were then dehydrated through two changes each of 95% and 100% EtOH and three changes of xylene before coverslipping in mounting media (Fisher Chemical, Thermo Fisher Scientific, Waltham, MA). Samples were left to dry overnight, and images were collected using a Leica microscope (DM2700 M, Leica, Buffalo Grove, IL).

### Simulation

A 2D axisymmetric model is built using COMSOL Multiphysics (version 5.5, COMSOL, Burlington, MA). The tissue is modeled as a multilayer composite with different properties and includes the skin (stratum corneum, epidermis, and dermis, treated as a single layer), fat layer, p.c., and the fascia. Dimensions and properties are specified to best represent the anatomy of rat skin and, in particular, that of our animal subjects. A model schematic and details are presented in the Supplementary Materials. A negative pressure is applied to the top layer of the skin model through a boundary load. A suction cup is included, and a pure slip of the skin surface is allowed underneath the cup rim to best mimic the real situation. The geometry is assumed to be axisymmetric about the cup axis. The tissue layers are mechanically isotropic, nearly incompressible, and hyperelastic. A neo-Hookean model is used for the skin, fat, and fascia layers, and an Ogden model is used for the muscle (p.c.) to best capture the different mechanical responses of the different tissue layers (*21*–*26*). The strain magnitude shown in the figures is defined as the Frobenius norm of the strain tensor, and the precise mathematical formula is found in the Supplementary Materials.

### Statistics

Statistical analysis is performed using Student’s *t* test for all figures: *P ≤ 0.05, **P ≤ 0.01, and ***P ≤ 0.001. Bars and error bars in Figs. 1 and 2 represent means ±SD. Bars and error bars in Fig. 4 represent geometric mean with 95% confidence interval. All statistics are generated using GraphPad Prism (9.0.0, GraphPad Software, San Diego, CA).

## Supplementary Materials

### This PDF file includes:

Supplementary Text Figs. S1 to S9 References

## Notes

View/request a protocol for this paper from *Bio-protocol*.
