# Supplementary material for: Novel suction-based in vivo cutaneous DNA transfection platform
Source: Sci Adv. 2021 Nov 5;7(45):eabj0611. doi: 10.1126/sciadv.abj0611 (PMC8570601; doi:10.1126/sciadv.abj0611)
Supplement: Supplementary file 1 — Supplementary Text Figs. S1 to S9 References [file sciadv.abj0611_sm.pdf]

Science Advances
AAAS

Supplementary Materials for

Novel suction-based in vivo cutaneous DNA transfection platform

Emran O. Lallow, Nandita C. Jhumur, Ijaz Ahmed, Sagar B. Kudchodkar, Christine C. Roberts, Moonsup Jeong, Juliet M. Melnik, Sarah H. Park, Kar Muthumani, Jerry W. Shan, Jeffrey D. Zahn, David I. Shreiber, Jonathan P. Singer, Young K. Park, Joel N. Maslow*, Hao Lin*

*Corresponding author. Email: jmaslow@genels.us (J.N.M.); hlin@soe.rutgers.edu (H.L.)

Published 5 November 2021, *Sci. Adv.* **7**, eabj0611 (2021)
DOI: 10.1126/sciadv.abj0611

The PDF file includes:

Supplementary Text
Figs. S1 to S9
References

Other Supplementary Material for this manuscript includes the following:

Movie S1

Supplementary Materials

Sectioned and 3D view of fluorescence expression

Skin was exercised at 24 hours post injection of pEGFP-N1 plasmids (25 μg in 50 μL 1x PBS solution) without or with subsequent suction treatment at 65 kPa for 30 s. For each condition (injection only or injection followed by application of suction) 6 samples of rat skin were excised. Each explanted skin sample was approximately 7.2 mm in diameter. Each skin sample was sectioned vertically to 60-µm-thick slices, creating a total of 120 x 12= 1440 slices, with every 5th slice stained and examined, totaling 24x12 = 288 slices such that GFP expression was determined at 300 µm intervals. Deeper penetration of the fluorescence was consistently observed for suction-treated cases, and representative images are shown in Fig. S1a and b. Furthermore, a 3D view of the expression intensity, generated using confocal microscopy per description in the Methods section, is demonstrated in Fig. S1c. Fig. S1d is a 3D x-z (side-view) demonstration of the same confocal data which also confirms the depth of the expression, namely, ~ 400 µm. The results are discussed in the proper text.

Raw intensity in the DNA concentration study

Figure S2 complement Fig. 2e and f in the main text, where the normalized intensity and expression area are shown. Here, the raw intensity shows a monotonic increase with respect to DNA concentration (or total DNA amount, as the injection volume remains the same), with or without suction treatment following injection. Note that raw intensities here are comparable as these experiments were performed on the same day with the same optical setting.

COMSOL simulation

A schematic of the model is shown in Fig. S3. The tissue is modeled as a multilayered soft composite including skin (stratum corneum, epidermis, and dermis, treated as a single 1.2-mm-thick layer), a 0.5-mm-thick fat layer, a 0.8-mm-thick muscle (panniculus carnosus, p.c.), and a 1 mm-thick fascia. Layer thickness was measured from histology images of adult male rat (SD) skin tissue to best mimic that of our animal subjects. Note that the anatomy is different to that of humans as the p.c. muscle is not present (*33, 34*). Tangential sliding (tearing) between the layers is not permitted. A cup is included, and a large Young's modulus of 3×10^9 Pa is prescribed which renders it effectively rigid. A negative pressure is applied to the top surface of the skin through a boundary load over the area covered by cup. The simulation geometry is axisymmetric about the cup axis. The skin and cup contact are modeled using a frictionless "contact pair" allowing to provide pure slip, which captures the realistic situation that skin slides into the cup under suction, with all skin that enters the cup receiving the boundary load. Fixed constraints are applied to the bottom surface

of the entire tissue model and the top surface of the cup, while all other surfaces are set to free boundary condition (Fig. S3). The simulation is static without considering the transient and viscoelastic effects. Because the model is incompressible, we have ignored any compressible effects including those induced by possible fluid transport within a tissue matrix that behaves like a porous medium.

For the different layers of our model, we use available constitutive model parameters from the literature. Among many hyperelastic models, we use the Neo-Hookean model which is the simplest one requiring only the Young's modulus and Poisson's ratio of the material. For skin, we use the Young's modulus of 130 kPa from Diridollou et al. where they also used a suction chamber to measure the vertical displacement of the skin's surface and incorporated a mathematical model of the mechanical behavior of a taught elastic membrane to obtain Young's modulus of the skin (*22*). For the other layers of our model, we use material properties obtained by other methods in the absence of suction. A shear modulus of 7.5 kPa of porcine fat measured by a rotational rheometer is used for the fat layer from Geerligs et al. (*23*), and the instantaneous tensile modulus of 4.77 kPa of *ex-vivo* rat subcutaneous tissue under uniaxial tension is used for fascia from Iatridis et al. (*24*). For the p.c. muscle, we use the available parameters for rat skeletal muscle under *in-vivo* compression from Bosboom et al. where they used comparatively high nonlinear Ogden model and their material parameters converged to stable values within 10 iterations independent of the chosen initial values (*26*). In our simplified model, the numerical simulation converged with the combination of these constitutive model parameters and the obtained simulation results match with our experimental results well. Some further details are below (*22*, *25*).

The Neo-Hookean strain energy function for a nearly incompressible material is (*21*):

$$W = \frac{E}{4(1+v)}(I_1 - 3) + \frac{E}{6\,(1-2v)}(J_{el} - 1)^2, \tag{S1}$$

where I_1 is the first invariant of elastic right Cauchy-Green tensor, J_{el} is the elastic volume ratio. The Young's modulus E is set to be 130 kPa (*22*), 22.2 kPa (*23*), and 4.77 kPa (*24*) for the skin, fat, and fascia layers, respectively. The Poisson's ratio is chosen to be 0.48 for all three layers (*25*).

The Ogden strain energy function to model muscle is:

$$W = \frac{\mu}{\alpha}(\lambda_1^\alpha + \lambda_2^\alpha + \lambda_3^\alpha - 3) + \frac{1}{2}k\,(J_{el} - 1)^2, \tag{S2}$$

where λ_i's are the principal stretches. The bulk and shear moduli, k and μ, are 384.8 kPa and 15.6 kPa, respectively. α is a material parameter and set to be 21.4 (*26*).

Figure S4 demonstrates the cross-sectional and top views of the von Mises stress under applied negative pressure of 65 kPa within the cup. While the top view corroborates with the expression pattern, the cross-sectional view reveals further details that the stress concentration of the ring pattern penetrates through the entire skin layer. Figure S5 and S6 demonstrate the strain and strain energy distributions which exhibit similar focal patterns; a 3D view of strain in the skin layer only has been presented in Fig. 4a in the proper text. Here the strain is computed as the Frobenius norm of the strain tensor, $\mathbf{E}$,

$$|\boldsymbol{E}| = \sqrt{\boldsymbol{E}:\boldsymbol{E}^T},$$

and the strain energy is

$$e = \frac{1}{2}\sigma : \boldsymbol{E},$$

where ":" denotes the inner product of tensors. Although both the fat and muscle layers experience significant stretching as well, they are not targeted for delivery in our studies.

Since skin is not a monolith and contains excess volume in wrinkles and dermal voids, it may be expected that the model would overestimate the local strain. Indeed, the predicted treatment size in the model due to slip is less than what is observed, indicative that the forces in the simulation are focused into a tighter region of material and thus magnified.

We also test deformation caused by the same condition on a human skin model, which has a slightly different anatomy where the p.c. muscle is absent. We use a 3-layer geometry with skin (2 mm), fat (8 mm), fascia (2 mm) to represent typical human anatomy (*34*, *35*); the model otherwise remains the same. We only show the strain in Figs. S7 and Fig. 3h in the proper text. Although the maximum strain in the human skin model appears to be several-folds higher, Fig. 3h indicates that this difference is primarily confined in a small region, whereas within the focal ring the overall strain magnitudes experienced are similar (0.25-1) in the skin layers despite the anatomic differences.

It would be of great interest to compare the area dilatational effect caused by strains produced with suction *in vivo* with the linear elongation strain used by Thottacherry et al. for single cells (*27*). Without losing generality, we assume that a cell is either spherical or ellipsoidal in its un-deformed, original shape with the semi-long and short axes denoted by a_0 and b_0, respectively. The spheroid/ellipsoid is then deformed to a new one denoted by a and b under a global strain, $|\mathbf{E}|$, while conserving volume, $ab^2 = a_0 b_0^2$. We have the relationship

$$|\boldsymbol{E}| = [(a/a_0 - 1)^2 + 2(b/b_0 - 1)^2]^{\frac{1}{2}}.$$

Meanwhile, we can compute the ellipsoidal surface area A and compare with the original area, denoted by A_0. The results are shown in Fig. S8a. A sphere is the global minimum in surface area, to reach just 10% area dilation requires a strain of $|\boldsymbol{E}|$ of approximately 0.8. On the other hand, relative area dilation increases as the original ellipsoid becomes more slender, indicated by increased aspect ratio of a_0/b_0. Fig. S8b demonstrates this effect. For an ellipsoid with initial aspect ratio of a_0/b_0 = 3, a 6% of linear strain $(a/a_0 - 1)$ would generate a commensurate area dilation of ~ 2.3%. This small dilatational effect will likely activate the CG pathway as indicated by Thottacherry et al. where typical cells are in elongated shape before stretching (*27*).

Histology results

The same skin sample as in Fig. 5 in the proper text is shown in Fig. S9 in a wider view.

Fig. S1. Photomicrograph of GFP expression in a sectioned view at 24 hours post DNA delivery.

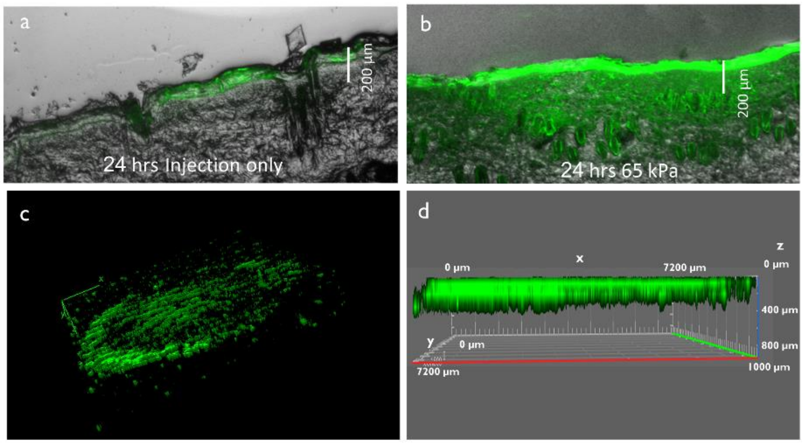

Figure S1: Fluorescence and confocal imaging of GFP expression of dorsal rat skin at 24 hrs post injection. a. DNA injection only. **b**. DNA injection followed by suction at 65 kPa for 30 s. **c**. 3D confocal image of GFP expression 24 hrs post injection and the application of 65 kPa for 30 s. **d**. An x-z view from the data of panel **c**, showing clearly a depth of expression around 400 μm from a side view.

Fig. S2. Raw fluorescence intensity with respect to DNA amount.

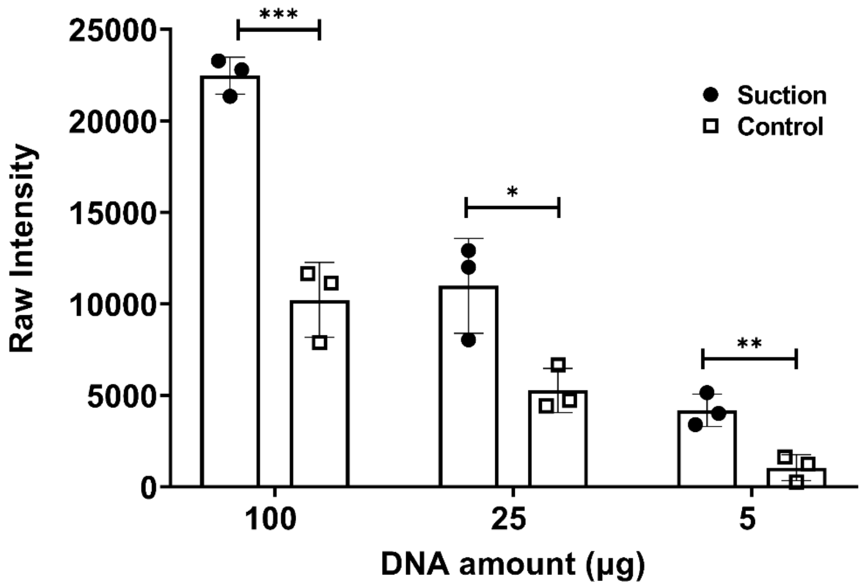

Figure S2: Raw fluorescence intensity with respect to DNA amount. The bars and error bars are mean and standard deviation, respectively. n=3 for each case. *, **, and *** represent $p \leq 0.05$, $p \leq 0.01$, and $p \leq 0.001$, respectively.

Fig. S3. A model schematic.

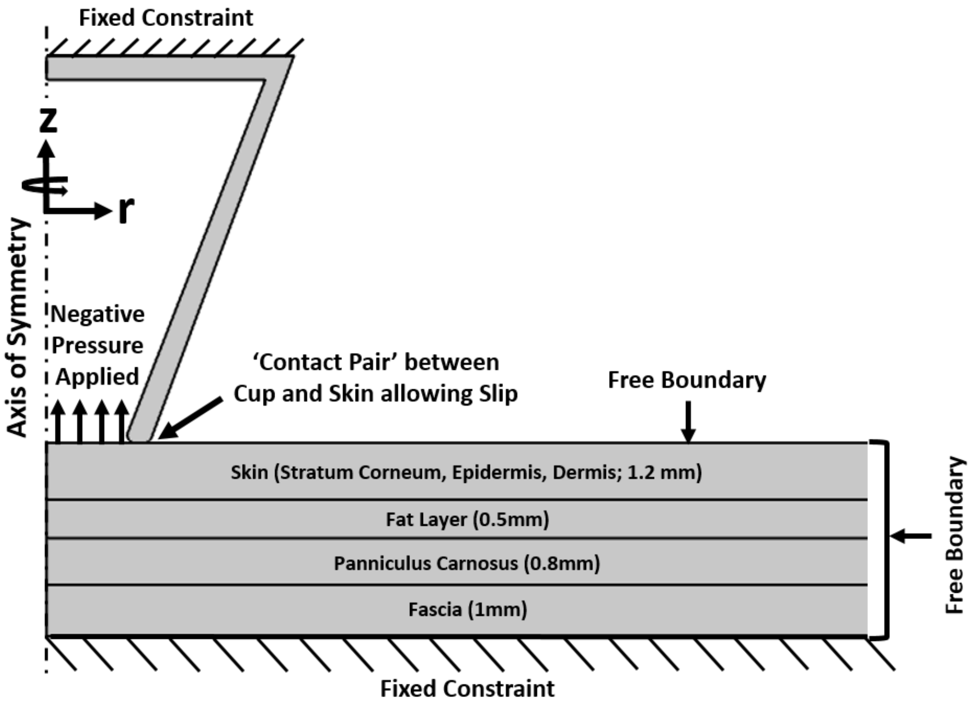

Figure S3: A model schematic (not to scale).

Fig. S4. Simulation results of Von Mises stress.

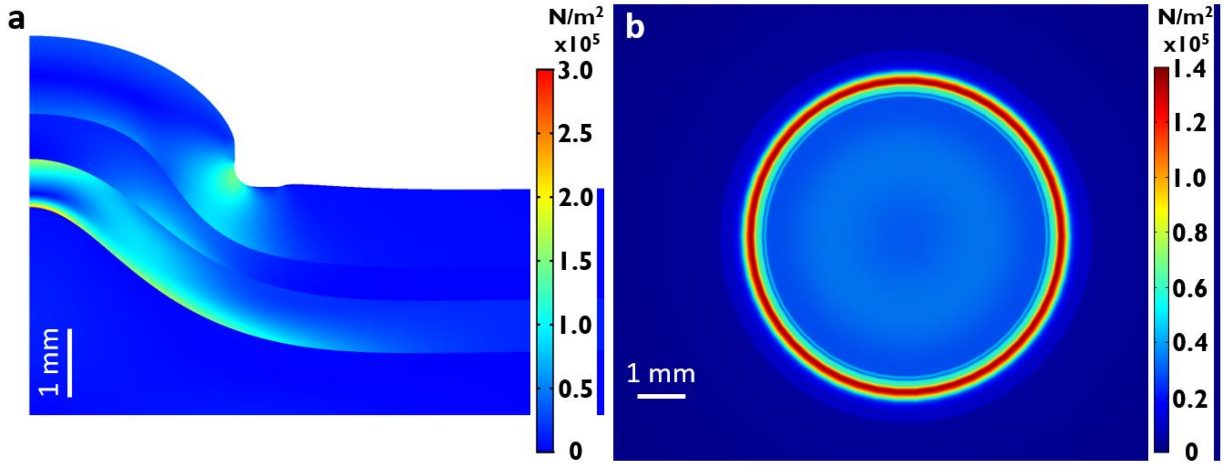

Figure S4: Simulation results of Von Mises stress. a. Cross-sectional and **b.** top view; the color map indicates magnitude of the von Mises stress. Pressure is 65 kPa.

Fig. S5. Simulation results of strain.

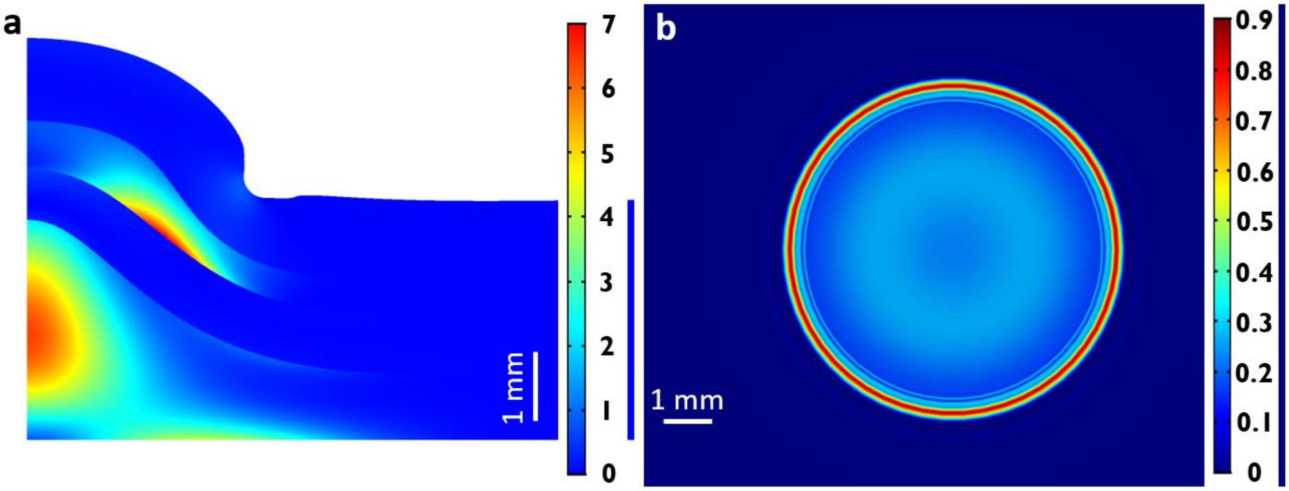

Figure S5: Simulation results of stain. a. Cross-sectional and **b.** top view; the color map indicates magnitude of the strain. Pressure is 65 kPa.

Fig. S6. Simulation results of strain energy.

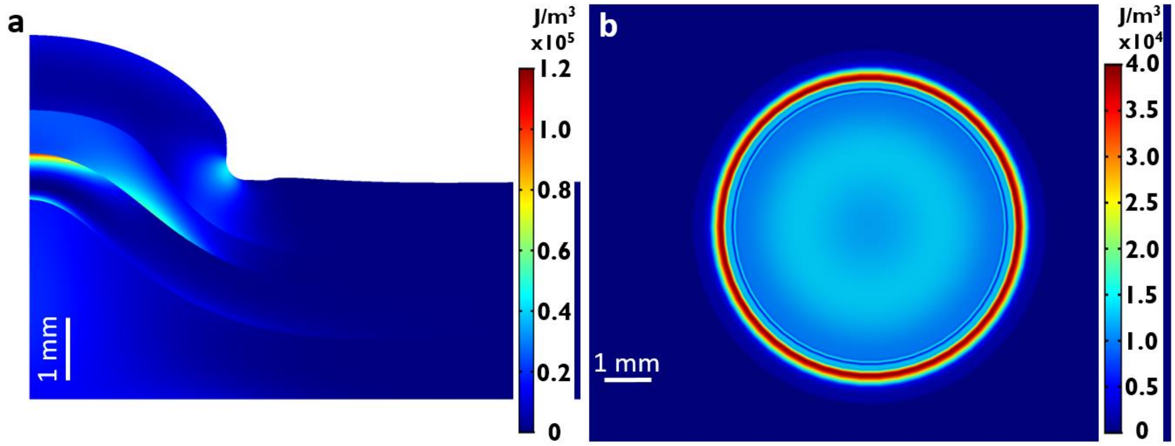

Figure S6: Simulation results of strain energy. a. Cross-sectional and **b.** top view; the color map indicates magnitude of the strain energy. Pressure is 65 kPa.

Fig. S7. Simulation results of human skin.

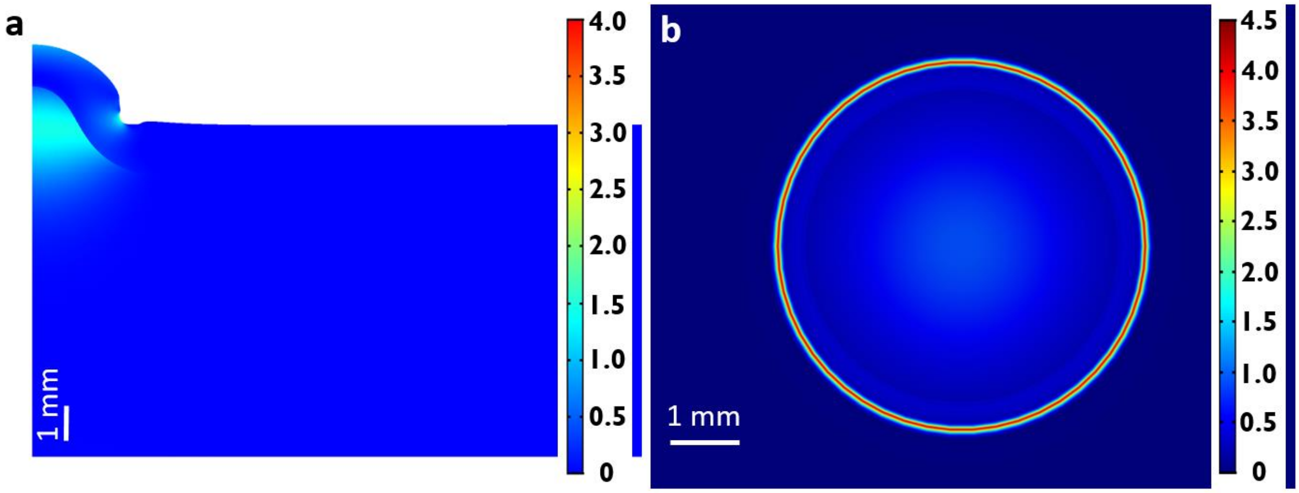

Figure S7: Simulation results of human skin. a. Cross-sectional and **b.** top view of a model modified to represent human skin with 2 mm, 8 mm, and 2 mm of skin, fat, and fascia, respectively; the color map indicates magnitude of the strain. Pressure is 65 kPa.

Fig. S8. Estimate of area dilation as a function of strain.

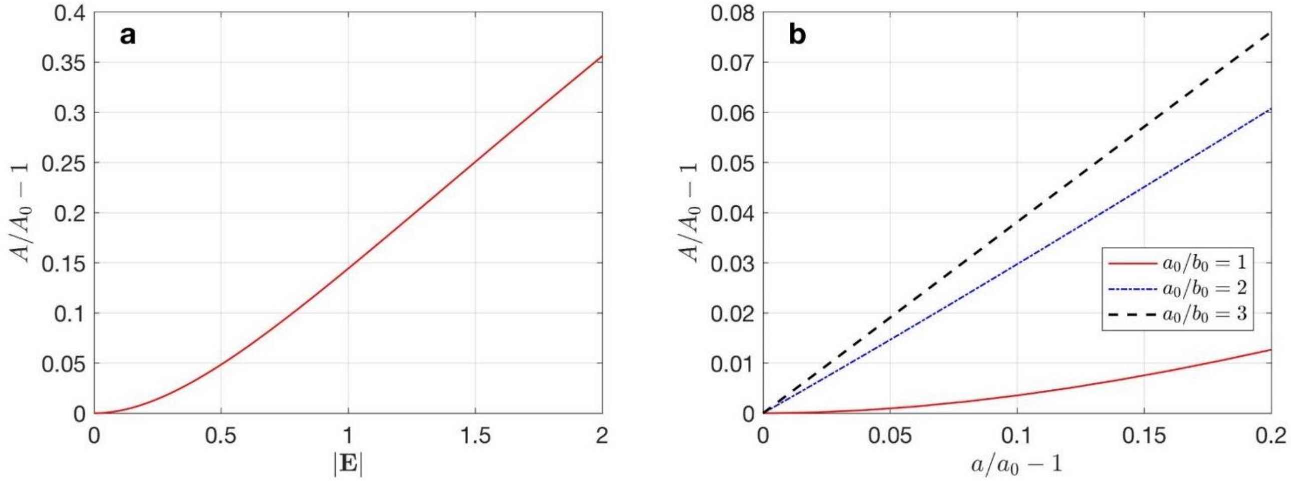

Figure S8: Area dilation as a function of stain. a. Estimate of area dilation as a function of strain (the Frobenius norm of the strain tensor) for a deformed spherical cell. **b.** Area dilation as a function of linear strain, $a/a_0 - 1$, for different starting aspect ratios of a_0/b_0.

Fig. S9. Histology via H&E staining.

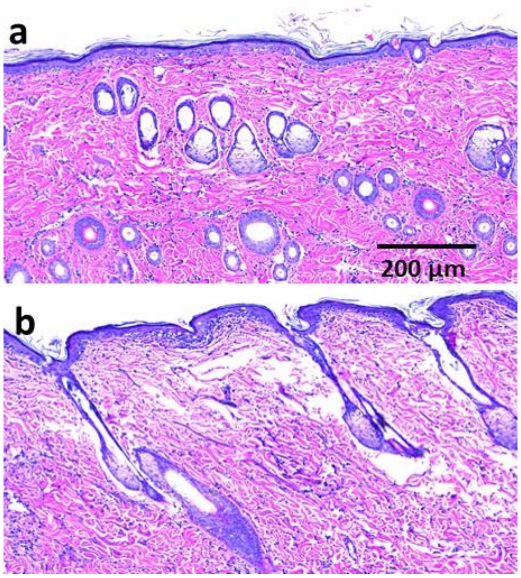

Figure S9: Histology via H&E staining. a. A section across the injection bleb from skin that received DNA injection only. **b.** A section of a skin area that received injection followed by suction.

REFERENCES AND NOTES

1. K. Sridharan, N. J. Gogtay, Therapeutic nucleic acids: Current clinical status. *Br. J. Clin. Pharmacol.* **82**, 659–672 (2016).

2. J. C. Burnett, J. J. Rossi, RNA-based therapeutics: Current progress and future prospects. *Chem. Biol.* **19**, 60–71 (2012).

3. T. T. Le, Z. Andreadakis, A. Kumar, R. G. Román, S. Tollefsen, M. Saville, S. Mayhew, The COVID-19 vaccine development landscape. *Nat. Rev. Drug Discov.* **19**, 305–306 (2020).

4. A. Kumar, T. S. Meldgaard, S. Bertholet, Novel platforms for the development of a universal influenza vaccine. *Front. Immunol.* **9**, 600 (2018).

5. C. B. Creech, S. C. Walker, R. J. Samuels, SARS-CoV-2 vaccines. *JAMA* **325**, 1318–1320 (2021).

6. I. Slivac, D. Guay, M. Mangion, J. Champeil, B. Gaillet, Non-viral nucleic acid delivery methods. *Expert Opin. Biol. Ther.* **17**, 105–118 (2017).

7. N. Pardi, M. J. Hogan, F. W. Porter, D. Weissman, mRNA vaccines—A new era in vaccinology. *Nat. Rev. Drug Discov.* **17**, 261- 279 (2018).

8. J. A. Ake, A. Schuetz, P. Pegu, L. Wieczorek, M. A. Eller, H. Kibuuka, F. Sawe, L. Maboko, V. Polonis, N. Karasavva, Safety and immunogenicity of PENNVAX-G DNA prime administered by Biojector 2000 or CELLECTRA electroporation device with modified vaccinia Ankara-CMDR boost. *J.Infect. Dis.* **216**, 1080–1090 (2017).

9. D. van Riel, E. de Wit, Next-generation vaccine platforms for COVID-19. *Nat. Mater.* **19**, 810–812 (2020).

10. K. Modjarrad, C. C. Roberts, K. T. Mills, A. R. Castellano, K. Paolino, K. Muthumani, E. L. Reuschel, M. L. Robb, T. Racine, M. D. Oh, C. Lamarre, F. I. Zaidi, J. Boyer, S. B. Kudchodkar, M. Jeong, J. M. Darden, Y. K. Park, P. T. Scott, C. Remigio, A. P. Parikh, M. C. Wise, A. Patel, E. K. Duperret, K. Y. Kim, H. Choi, S. White, M. Bagarazzi, J. M. May, D. Kane, H. Lee, G. Kobinger, N. L. Michael, D. B. Weiner, S. J. Thomas, J. N. Maslow, Safety and immunogenicity of an anti-Middle East respiratory syndrome coronavirus DNA vaccine: A phase 1, open-label, single-arm, dose-escalation trial. *Lancet Infect. Dis.* **19**, 1013–1022 (2019).

11. M. R. Gaudinski, K. V. Houser, K. M. Morabito, Z. Hu, G. Yamshchikov, R. S. Rothwell, N. Berkowitz, F. Mendoza, J. G. Saunders, L. Novik, Safety, tolerability, and immunogenicity of two Zika virus DNA vaccine candidates in healthy adults: Randomised, open-label, phase 1 clinical trials. *Lancet* **391**, 552–562 (2018).

12. M. Wallace, B. Evans, S. Woods, R. Mogg, L. Zhang, A. C. Finnefrock, D. Rabussay, M. Fons, J. Mallee, D. J. M. T. Mehrotra, Tolerability of two sequential electroporation treatments using MedPulser DNA delivery system (DDS) in healthy adults. *Mol. Ther.* **17**, 922–928 (2009).
13. K. Schultheis, T. R. F. Smith, W. B. Kiosses, K. A. Kraynyak, A. Wong, J. Oh, K. E. Broderick, Delineating the cellular mechanisms associated with skin electroporation. *Hum. Gene Ther. Methods* **29**, 177–188 (2018).
14. P. Tebas, C. C. Roberts, K. Muthumani, E. L. Reuschel, S. B. Kudchodkar, F. I. Zaidi, S. White, A. S. Khan, T. Racine, H. Choi, Safety and immunogenicity of an anti–Zika virus DNA vaccine—Preliminary report. *New Engl. J. Med.* **385**, e35 (2017).
15. J. Hooper, K. M. Paolino, K. Mills, S. Kwilas, M. Josleyn, M. Cohen, B. Somerville, M. Wisniewski, S. Norris, B. Hill, M. Sanchez-Lockhart, D. Hannaman, C. S. Schmaljohn, A phase 2a randomized, double-blind, dose-optimizing study to evaluate the immunogenicity and safety of a bivalent DNA vaccine for hemorrhagic fever with renal syndrome delivered by intramuscular electroporation. *Vaccines* **8**, 377 (2020).
16. A. M. Al-Bedah, I. S. Elsubai, N. A. Qureshi, T. S. Aboushanab, G. I. Ali, A. T. El-Olemy, A. A. Khalil, M. K. Khalil, M. S. Alqaed, The medical perspective of cupping therapy: Effects and mechanisms of action. *J. Tradit. Complement. Med.* **9**, 90–97 (2019).
17. H. Cao, X. Li, J. Liu, An updated review of the efficacy of cupping therapy. *PLOS ONE* **7**, e31793 (2012).
18. K. Shimizu, S. Kawakami, K. Hayashi, H. Kinoshita, K. Kuwahara, K. Nakao, M. Hashida, S. J. P. O. Konishi, In vivo site-specific transfection of naked plasmid DNA and siRNAs in mice by using a tissue suction device. *PLOS ONE* **7**, e41319 (2012).
19. Y. Taniguchi, N. Oyama, S. Fumoto, H. Kinoshita, F. Yamashita, K. Shimizu, M. Hashida, S. Kawakami, Tissue suction-mediated gene transfer to the beating heart in mice. *PLOS ONE* **15**, e0228203 (2020).
20. D. H. Amante, T. R. Smith, J. M. Mendoza, K. Schultheis, J. R. McCoy, A. S. Khan, N. Y. Sardesai, K. E. Broderick, Skin transfection patterns and expression kinetics of electroporation-enhanced plasmid delivery using the CELLECTRA-3P, a portable next-generation dermal electroporation device. *Hum. Gene Ther. Methods* **26**, 134–146 (2015).
21. A. F. Bower, *Applied Mechanics of Solids* (CRC Press, 2009).

22. S. Diridollou, F. Patat, F. Gens, L. Vaillant, D. Black, J. Lagarde, Y. Gall, M. Berson, In vivo model of the mechanical properties of the human skin under suction. *Skin Res. Techol.* **6**, 214–221 (2000).
23. M. Geerligs, G. W. Peters, P. A. Ackermans, C. W. Oomens, F. Baaijens, Linear viscoelastic behavior of subcutaneous adipose tissue. *J. Biorheol.* **45**, 677–688 (2008).
24. J. C. Iatridis, J. Wu, J. A. Yandow, H. M. Langevin, Subcutaneous tissue mechanical behavior is linear and viscoelastic under uniaxial tension. *Connect. Tissue Res.* **44**, 208–217 (2003).
25. C. Li, G. Guan, R. Reif, Z. Huang, R. K. Wang, Determining elastic properties of skin by measuring surface waves from an impulse mechanical stimulus using phase-sensitive optical coherence tomography. *J. R. Soc. Interface* **9**, 831–841 (2012).
26. E. Bosboom, M. Hesselink, C. Oomens, C. Bouten, M. Drost, F. Baaijens, Passive transverse mechanical properties of skeletal muscle under in vivo compression. *J. Biomech.* **34**, 1365–1368 (2001).
27. J. J. Thottacherry, A. J. Kosmalska, A. Kumar, A. S. Vishen, A. Elosegui-Artola, S. Pradhan, S. Sharma, P. P. Singh, M. C. Guadamillas, N. Chaudhary, R. Vishwakarma, X. Trepat, M. A. Del Pozo, R. G. Parton, M. Rao, P. Pullarkat, P. Roca-Cusachs, S. Mayor, Mechanochemical feedback control of dynamin independent endocytosis modulates membrane tension in adherent cells. *Nat. Commun.* **9**, 4217 (2018).
28. M. Mao, L. Wang, C. C. Chang, K. E. Rothenberg, J. Huang, Y. Wang, B. D. Hoffman, P. B. Liton, F. Yuan, Involvement of a Rac1-dependent macropinocytosis pathway in plasmid DNA delivery by electrotransfection. *Mol. Ther.* **25**, 803–815 (2017).
29. C.-C. Chang, M. Wu, F. Yuan, Role of specific endocytic pathways in electrotransfection of cells. *Mol. Ther. Methods Clin. Dev.* **1**, 14058 (2014).
30. L. Wang, S. E. Miller, F. Yuan, Ultrastructural analysis of vesicular transport in electrotransfection. *Microsc. Microanal.* **24**, 553–563 (2018).
31. F. Lin, X. Shen, G. Kichaev, J. M. Mendoza, M. Yang, P. Armendi, J. Yan, G. P. Kobinger, A. Bello, A. S. Khan, Optimization of electroporation-enhanced intradermal delivery of DNA vaccine using a minimally invasive surface device. *Hum. Gene Ther. Part B Methods* **23**, 157–168 (2012).
32. L. D. Cervia, F. Yuan, Current progress in electrotransfection as a nonviral method for gene delivery. *Mol. Pharm.* **15**, 3617–3624 (2018).

33. N. Naldaiz-Gastesi, O. A. Bahri, A. Lopez de Munain, K. J. McCullagh, A. Izeta, The panniculus carnosus muscle: An evolutionary enigma at the intersection of distinct research fields. *J. Anat.* **233**, 275–288 (2018).
34. L. M. Tham, H. P. Lee, C. Lu, Cupping: From a biomechanical perspective. *J. Biomech.* **39**, 2183–2193 (2006).
35. H. K. Hong, Y. C. Jo, Y. S. Choi, H. D. Park, B. J. Kim, *SENSORS*, *2009 IEEE* (IEEE, 2009), pp. 695–698.
